# 

**Published:** 1904-02-06

**Authors:** 


					J. J-LJld J-L l/Ol JLJ.JT1.jLJ.
CADBURY'S U tot CADBURY'S
COCOA JL MM Jgg_r COCOA
"A PERFECT FOOD." NO DRUGS OR ALKALiua im>h, ,
s >?
.No. 906. Vol. XXXV. [Ktfl Saturday,
Feb. 6th, 1904.
[Eiubseription Tj.erm?,J pRICE THREEPENCE.

				

